# Deletion of the Ca^2+^ Channel Subunit α_2_δ3 Differentially Affects Ca_v_2.1 and Ca_v_2.2 Currents in Cultured Spiral Ganglion Neurons Before and After the Onset of Hearing

**DOI:** 10.3389/fncel.2019.00278

**Published:** 2019-06-26

**Authors:** Friederike Stephani, Veronika Scheuer, Tobias Eckrich, Kerstin Blum, Wenying Wang, Gerald J. Obermair, Jutta Engel

**Affiliations:** ^1^Department of Biophysics, Center for Integrative Physiology and Molecular Medicine, School of Medicine, Saarland University, Homburg, Germany; ^2^Department of Physiology, School of Medicine, University of Nevada, Reno, NV, United States; ^3^Department of Physiology and Medical Physics, Medical University Innsbruck, Innsbruck, Austria; ^4^Division Physiology, Karl Landsteiner University of Health Sciences, Krems, Austria

**Keywords:** Ca^2+^ channel, Ca^2+^ current, P/Q-type, N-type, postnatal development, auditory, auxiliary subunit, primary culture

## Abstract

Voltage-gated Ca^2+^ channels are composed of a pore-forming α_1_ subunit and auxiliary β and α_2_δ subunits, which modulate Ca^2+^ current properties and channel trafficking. So far, the partial redundancy and specificity of α_1_ for α_2_δ subunits in the CNS have remained largely elusive. Mature spiral ganglion (SG) neurons express α_2_δ subunit isoforms 1, 2, and 3 and multiple Ca^2+^ channel subtypes. Differentiation and *in vivo* functions of their endbulb of Held synapses, which rely on presynaptic P/Q channels (Lin et al., 2011), require the α_2_δ3 subunit (Pirone et al., 2014). This led us to hypothesize that P/Q channels may preferentially co-assemble with α_2_δ3. Using a dissociated primary culture, we analyzed the effects of α_2_δ3 deletion on somatic Ca^2+^ currents (*I_Ca_*) of SG neurons isolated at postnatal day 20 (P20), when the cochlea is regarded to be mature. P/Q currents were the dominating steady-state Ca^2+^ currents (54% of total) followed by T-type, L-type, N-type, and R-type currents. Deletion of α_2_δ3 reduced P/Q- and R-type currents by 60 and 38%, respectively, whereas L-type, N-type, and T-type currents were not altered. A subset of *I_Ca_* types was also analyzed in SG neurons isolated at P5, i.e., before the onset of hearing (P12). Both L-type and N-type current amplitudes of wildtype SG neurons were larger at P5 compared with P20. Deletion of α_2_δ3 reduced L-type and N-type currents by 23 and 44%, respectively. In contrast, small P/Q currents, which were just being up-regulated at P5, were unaffected by the lack of α_2_δ3. In summary, α_2_δ3 regulates amplitudes of L- and N-type currents of immature cultured SG neurons, whereas it regulates P/Q- and R-type currents at P20. Our data indicate a developmental switch from dominating somatic N- to P/Q-type currents in cultured SG neurons. A switch from N- to P/Q-type channels, which has been observed at several central synapses, may also occur at developing endbulbs of Held. In this case, reduction of both neonatal N- (P5) and more mature P/Q-type currents (around/after hearing onset) may contribute to the impaired morphology and function of endbulb synapses in α_2_δ3-deficient mice.

## Introduction

Voltage-gated calcium channels (VGCCs) consist of a pore-forming α_1_ subunit and auxiliary β and α_2_δ subunits (Catterall et al., 2005; Dolphin, 2012; Zamponi et al., 2015). The extracellular α_2_δ proteins assist in trafficking and proper surface expression of the channel complex (Catterall, 2000; Dolphin, 2012, 2013, 2018). In heterologous expression systems, any α_1_ subunit can co-assemble with any of the four α_2_δ subunits, but in native systems there are partially specific functions for particular α_2_δ proteins (Dolphin, 2012, 2013, 2018; Geisler et al., 2015, 2019). The reasons for this partial specificity are so far unclear. Isoforms α_2_δ1–3 are widely expressed in the brain and often co-expressed in the same type of neuron (Cole et al., 2005; Schlick et al., 2010; Geisler et al., 2015). Recently, additional functions have been found for particular α_2_δ subunits including channel trafficking along axons, synapse development, and trans-synaptic alignment (Eroglu et al., 2009; Kurshan et al., 2009; Fell et al., 2016; Kadurin et al., 2016; Dolphin, 2018; Ferron et al., 2018; Geisler et al., 2019).

Cochlear spiral ganglion (SG) neurons transmit sound-evoked information transduced by hair cells to the brain. They consist of 95% myelinated type I neurons innervating inner hair cells (IHC) and 5% unmyelinated type II neurons integrating information from multiple outer hair cells. Each of the 10–20 ribbon synapses of one IHC is innervated by a type I SG neuron in a 1:1 manner. The axon of each SG neuron, which represents one fiber of the auditory nerve, branches and forms synapses at multiple targets in the cochlear nuclear complex in the brainstem (Malmierca and Merchán, 2004; Rusznak and Szucs, 2009). Bushy cells (BC) in the anteroventral cochlear nucleus receive large axosomatic synapses from auditory nerve fibers called endbulbs of Held (Ryugo, 1992; Limb and Ryugo, 2000), and each BC receives input from 5–7 SG neurons as shown for rat (Nicol and Walmsley, 2002). These glutamatergic excitatory synapses operate at high rates and with utmost temporal precision to preserve temporal information of sound signals (Joris et al., 1994). Glutamate release at the juvenile endbulb synapse is triggered by Ca^2+^ influx, which is largely (85% of total) flowing through P/Q (Ca_v_2.1) channels (Oleskevich and Walmsley, 2002; Lin et al., 2011).

Current knowledge about ion currents in SG neurons of hearing mice is sparse because these neurons are housed in the bony middle axis of the ossified cochlea, preventing slice recordings. Moreover, their soma is completely covered by the soma of satellite cells and their myelin from P0 onward in mice (Wang et al., 2013), which prevents recordings from acutely dissected SG tissue and requires dissociated primary cultures (Lee et al., 2016). Primary cultured SG neurons from 3-month-old mice express a variety of voltage-gated Ca^2+^ channels since L-, P/Q-, N-, R-, and T-type currents have been identified by whole cell and single channel recordings (Lv et al., 2012, 2014). Single cell RNA sequencing confirmed transcripts for these Ca_v_ channels and revealed α_2_δ1, α_2_δ2, and α_2_δ3 but not α_2_δ4 transcripts in SG neurons of 4-week-old mice (Shrestha et al., 2018). Because of strong glycosylation of α_2_δ proteins, labeling them with antibodies is challenging and to date no specific antibodies exist to label α_2_δ3 protein in tissue at the cellular and subcellular level.

Previously, we have shown that lack of α_2_δ3 in mice causes impaired *in vivo* function of the endbulb of Held synapse resulting in an auditory processing disorder (Pirone et al., 2014). Endbulb synapses are smaller and malformed in 5-week-old α_2_δ3^–/–^ mice. The observed reduction in auditory evoked input-output functions of the endbulb synapse suggests that impaired synaptic transmission may be caused by reduced presynaptic Ca^2+^ currents, malformed endbulbs of Held or both. A reduced number of Ca_v_2.1-immunolabeled puncta at SG neuron somata of 5-week-old α_2_δ3^–/–^ mice (Pirone et al., 2014) is in line with a preference of P/Q channels for α_2_δ3 and may suggest that presynaptic endbulb Ca^2+^ currents are reduced, too. Unfortunately, Ca^2+^ current recordings from tiny α_2_δ3^–/–^ endbulbs of Held are not feasible. Because presynaptic Ca^2+^ channels need to be synthetized and trafficked to synapses, we aimed at defining whether genetic ablation of α_2_δ3 differentially alters the composition of somatic voltage-activated Ca^2+^ currents in primary cultured SG neurons of 3-week-old wildtype and α_2_δ3^–/–^ mice, 8 days after the onset of hearing at P12 (Ehret, 1985). We also studied the presence of *Cacna2d3* (α_2_δ3) transcripts and characterized a subset of voltage-activated Ca^2+^ currents in SG neurons of pre-hearing (P5) mice to ascertain whether neonatal L-type (Ca_v_1.2/Ca_v_1.3) channels as well as Ca_v_2.1 and Ca_v_2.2 channels require α_2_δ3 subunits at this young age.

## Materials and Methods

### Animals

All experimental procedures were conducted in agreement with the European Communities Council Directive (2010/63/EU) in accordance with the German law and the regional board for scientific animal experiments of the Saarland. Additional ethics approval was not required according to the local and national guidelines. Prehearing and hearing mice of either sex were studied. *Cacna2d3*-deficient mice generated by Deltagen (B6.129P2-Cacna2d3^tm1Dgen^, San Mateo, CA, United States) (Neely et al., 2010) were obtained from the Jackson Laboratories, back-crossed on C57Bl6/N background for ≥10 generations and used for electrophysiological and immunohistological analysis. Knockout was obtained by targeted insertion of a bacterial LacZ cassette into exon 15 (of 39) of the *Cacna2d3* gene such that the endogenous promoter drives the expression of β-galactosidase and of a truncated *Cacna2d3* mRNA (exons 1–14) (Neely et al., 2010). Cochleae were dissected after mice had been sacrificed with isoflurane anesthesia and cervical dislocation (P19–P21, denoted as P20) or by decapitation (P4–P6, denoted as P5). Animals were housed with free access to food and water at 22°C and a 12 h light-dark cycle. Every experimental observation was confirmed in at least three experiments using different animals.

### Dissociated Primary Culture of SG Neurons

SG neurons were isolated from neonatal (P5) and juvenile mice (P20) (Lee et al., 2016). To allow proper attachment of the isolated neurons, coverslips with a diameter of 13 mm were coated with poly-D-lysine (0.5 mg/ml) for 7 h at 37°C and overnight with laminin (1 mg/ml; both Sigma-Aldrich, St. Louis, MO, United States). The cell culture medium was prepared as follows: Neurobasal A medium (Invitrogen, Carlsbad, CA, United States) was supplemented with B27 (2% v/v), L-glutamine (200 mM, Invitrogen, Carlsbad, CA, United States), and penicillin G (100 U/μl, Sigma-Aldrich, St. Louis, MO, United States). The dissection solution contained 50 ml minimal essential medium (MEM, Invitrogen, Carlsbad, CA, United States), 400 μl MgCl_2_ (stock solution 1 M), 110 μl D-glucose (450 mg/ml), and 50 μl penicillin G (100 U/μl). The solution was mixed and 2 ml were taken away to prepare the digestion solution, supplemented with B27 (2% v/v). 2 ml FBS (Invitrogen, Carlsbad, CA, United States) were added to the dissection solution and mixed. To prepare the centrifugation solution, 3 ml of the dissection solution was mixed with the same volume of solution 2, consisting of 25 ml 10× HBSS (Invitrogen, Carlsbad, CA, United States) and 154 g sucrose, final volume 500 ml, pH 7.5. For a primary culture of P20 SG neurons, tissue of four mice (eight cochleae) of the same genotype was pooled. For neonatal mice, tissue from three mice (six cochleae) was sufficient. After decapitation, the head was sagitally cut into halves and stored in ice-cold MEM. The cartilage-like (P5) or bony shell (P20) of the cochlea was carefully removed and the cochlear spiral was separated into an apical and a basal half in dissection solution under sterile conditions (Lee et al., 2016). The halves were transferred into petri dishes filled with 1 ml digestion solution. Tissue pieces were disintegrated and affiliated in 400 μl digestion solution in a 2 ml Eppendorf tube each. The tissue was incubated with 50 μl DNase I (1000 U/ml, Sigma-Aldrich, St. Louis, MO, United States) and 50 μl collagenase type I (10 mg/ml, Sigma-Aldrich, St. Louis, MO, United States) in a 37°C water bath for 15 min, followed by adding 1 μl 0.25 M NaOH and 50 μl 2.5% trypsin (Invitrogen, Carlsbad, CA, United States) and shaking for 10 min at 37°C at 180/min. To stop the enzymatic digestion, 400 μl FBS (Invitrogen, Carlsbad, CA, United States) was added; remainders of the tissue were carefully triturated and 800 μl centrifugation solution was added. Cells were centrifuged at room temperature for 5 min at 845 *g*. The supernatant was aspirated and the cell pellet was dissolved in 400 μl pre-warmed cell culture medium. After carefully re-suspending the pellet and filtering through a 40 μm cell strainer (Fisher Scientific, Hampton, NH, United States), 400 μl of cell suspension was plated onto two wells (400 μl/well) supplemented with 1 μl NT3 and 1 μl BDNF (both Sigma-Aldrich, St. Louis, MO, United States) at 10 ng/μl, respectively. When SG neurons were isolated from mature mice, 40 μl FBS was added. SG neurons were placed into an incubator at 37°C and 5% CO_2_. The medium was replaced by medium supplemented with 10 ng/μl BDNF and NT3 each by 100% after 1 day and by 50% on the following days. SG neurons isolated at P5 were cultured for 2 days, SG neurons isolated at P20 were cultured for 3 days.

### Electrophysiology

Currents from SG neurons of α_2_δ3^+/+^ and α_2_δ3^–/–^ mice were recorded using an Axopatch 200B patch clamp amplifier (Molecular Devices, Sunnyvale, CA, United States) with PatchMaster V2x69 (Heka Electronics, Lambrecht, Germany) at room temperature (21 ± 1°C). The bath solution consisted of (in mM): 1.3 CaCl_2_, 10 HEPES, 5.6 glucose, 5.8 KCl, 0.9 MgCl_2_, 143 NaCl and 0.9 NaH_2_PO_4_ × H_2_O, pH 7.35, 305 mosmol/kg. To isolate Ca^2+^ currents, cells were locally superfused with application solution (in mM): 15 4-AP, 1.3 CaCl_2_, 2 CsCl, 5.6 glucose, 10 HEPES, 1 MgCl_2_, 0.7 NaH_2_PO_4_ × H_2_O, 30 TEA-Cl, 113 NMDG, pH 7.35, 305 mosmol/kg, via a custom-made gravity-fed application system. The pipette solution consisted of (in mM): 0.1 CaCl_2_, 20 CsCl, 5 EGTA, 0.3 GTP, 5 HEPES acid, 4 MgCl_2_, 4 Na_2_ATP, 10 Na-phosphocreatine, 110 Cs-methanesulfonate, 295 mosmol/kg, pH was adjusted to 7.35 using 1 M CsOH.

For each recording involving application of a Ca^2+^ channel blocker, a new piece of the glass coverslip with cultured SG neurons was used that had been carefully cracked into pieces with the blunt end of a forceps. Patch pipettes were pulled from quartz glass with a laser micropipette puller P-2000 (Sutter Instruments, Novato, CA, United States) with a resistance of 6–8 MΩ. Cells with a large, round soma and one or two neurites were selected. After establishing the whole cell mode, neuron identity was confirmed by large and fast inactivating inward (Na^+^) currents, which were blocked after perfusion with application solution for 1 min, giving rise to smaller slowly inactivating inward Ca^2+^ currents. Recordings were accepted when the membrane resistance was >1 GΩ. SG neurons were held at nominally −80 mV. Series resistance usually was between 5 and 15 MΩ, recordings with a larger series resistance were rejected. No compensation for the series resistance was employed because of relatively small Ca^2+^ currents. Data acquisition rate was 10 kHz, currents were filtered at 2 kHz.

L-, P/Q-, N-, R-, and T-type Ca^2+^ currents were isolated with 10 μM nimodipine (Sigma-Aldrich, St. Louis, MO, United States), 1 μM ω-agatoxin IVA, 1 μM ω-conotoxin, 1 μM SNX482 (from Alomone Labs, Jerusalem, Israel) and 5 μM mibefradil (Tocris Bioscience, Bristol, United Kingdom), respectively. Toxins were dissolved at 1 mM in distilled H_2_O and stored in aliquots at −20°C until use. Mibefradil and nimodipine were dissolved at 5 and 10 mM in DMSO, respectively, and stored at −20°C. During application of the blocker we observed run-up of *I_Ca_* in 8% of the recordings, resulting in negative difference currents. All data from these neurons were omitted from the analysis. Measurements of a particular Ca^2+^ current subtype of a given genotype and age included recordings of SG neurons from 3 to 5 (average: 3.6) culture preparations.

### Analysis of Patch Clamp Data

Patch clamp data were analyzed using the software Igor Pro Version 6.12 (Wavemetrics, Lake Oswego, OR, United States) and custom-written routines. Linear leak subtraction was done off-line and voltages were corrected by subtracting a liquid junction potential (LJP) of 27.8 mV for the three-solution setting (Neher, 1992). Steady-state current-voltage (*I/V*) relations were calculated by averaging *I_Ca_* during the last ms of the 100 ms depolarizing pulse. Voltage-gated Ca^2+^ currents (*I_Ca_*) of a given genotype were variable within SG neurons from a particular cochlear location but after statistical tests did not show systematic changes between apical or basal halves. Therefore, *I_Ca_* data were pooled for the whole cochlear length for a given genotype and age.

### Quantitative Real-Time PCR for *Cacna2d1*, *Cacna2d2*, and *Cacna2d3* in SG Tissue

For qPCR analysis, spiral ganglia from α_2_δ3^–/–^ and α_2_δ3^+/–^ mice aged P5 were microdissected and separated into apical and basal halves. Because heterozygous α_2_δ3^+/–^ mice do not show a phenotype they served as controls for qPCR experiments (Neely et al., 2010). Pooled apical and basal SG from two animals each were transferred into cryotubes, frozen in liquid nitrogen and stored at −70°C. Total RNA was isolated using the peqGOLD MicroSpin Total RNA Kit (PeqLab Biotechnologie GmbH, Erlangen, Germany) according to the protocol of the manufacturer. For reverse transcription (RT), isolated RNA (10 μl each) was incubated with 0.5 μl oligo dT20 primers (50 mM) and random primers pd(N)_6_ (50 μM, Applied Biosystems, Carlsbad, CA, United States) and 1 μl dNTP mix (10 mM; New England Biolabs, Ipswich, MA, United States) for 5 min at 65°C and stored on ice for 1 min.

An RT mix (8 μl; Life Technologies, Carlsbad, CA, United States) consisting of 4 μl 5× RT buffer, 2 μl dithiothreitol (100 mM), 1 μl RNaseOUT^TM^ and 1 μl SuperScript® III was added and each tube was incubated at 50°C for 150 min followed by 70°C for 30 min. cDNA was stored at −20°C. The abundance of *Cacna2d1–3* transcripts in SG cDNA was assessed by TaqMan quantitative PCR (qPCR) using a standard curve method (Schlick et al., 2010). TaqMan gene expression assays specific for *Cacna2d1–3* isoforms were designed to span exon–exon boundaries and purchased from Applied Biosystems. The following assays were used [name (gene symbol), assay ID (Applied Biosystems)]: α_2_δ1 (*Cacna2d1*), Mm00486607_m1; α_2_δ2 (*Cacna2d2*), Mm00457825_m1; α_2_δ3 (*Cacna2d3*), Mm00486613_m1; α_2_δ4 (*Cacna2d4*), Mm01190105_m1. Expression of hypoxanthine phosphoribosyl-transferase 1 (*Hprt1*; Mm00446968_m1) and succinate dehydrogenase, subunit A (*Sdha*; Mm01352363_m1) were used as endogenous controls. The qPCR (50 cycles) was performed in duplicates using total DNA (see above) and the specific TaqMan gene expression assay for each 20 μl reaction in TaqMan Universal PCR Master Mix (Applied Biosystems, Foster City, CA, United States). Samples without cDNA were used as controls. Analyses were performed using the 7500 Fast System (Applied Biosystems, Foster City, CA, United States). The *Ct* values for each gene expression assay were recorded for each individual preparation and molecule numbers were calculated for each α_2_δ subunit from their respective standard curve. Expression of *Hprt1* and *Sdha* were used for normalization of total mRNA abundance to allow a direct comparison between the expression levels in different preparations.

### Immunohistochemistry

SG tissue from α_2_δ3^+/+^ and α_2_δ3^–/–^ mice was dissected after perfusion of the cochlea with Zamboni’s fixative (Stefanini et al., 1967) for 15 min, mounted on coverslips and double-labeled with rabbit polyclonal anti-Ca_v_2.1 (Synaptic Systems, Göttingen, Germany, #152203/7, 1:500) and mouse monoclonal anti-Golgi matrix protein 130 kDa (GM 130, BD Transduction Laboratories, San Jose, CA, United States, #610823, 1:50) as described (Fell et al., 2016). Donkey anti-mouse Alexa Fluor® 488 (Invitrogen, Carlsbad, CA, United States, #A-21202, 1:500) and donkey anti-rabbit Cy3 (Jackson Immuno, West Grove, PA, United States, #711-166-152, 1:1500) were used as secondary antibodies. Images were acquired with a confocal microscope LSM 710 using a 63×/1.4 NA oil objective (both Zeiss Microscopy GmbH, Göttingen, Germany). Single optical slices with a thickness of 0.31 μm are shown.

### Statistical Analysis

Igor Pro Version 6.12 (Wavemetrics, Lake Oswego, OR, United States) was used for statistical tests of *I_Ca_*. Because often data were not normally distributed, box plots and individual data points are shown, with boxes representing the 25th–75th percentiles, the median (horizontal bar) and the 10th–90th percentiles (whiskers). Comparisons between *I_Ca_* without/with blocker (paired samples) were performed using the Wilcoxon signed test, comparisons of unpaired samples using the Wilcoxon rank test. Transcript data were analyzed on log10-transformed transcript numbers using two-way ANOVA with Holm–Sidak *post hoc* correction. Electrophysiological data are given as mean ± SD, transcript data as mean ± SEM.

## Results

### L-Type Ca^2+^ Currents Were Unaltered in Cultured SG Neurons of 3-Week-Old α_2_δ3^–/–^ Mice

**FIGURE 1:**
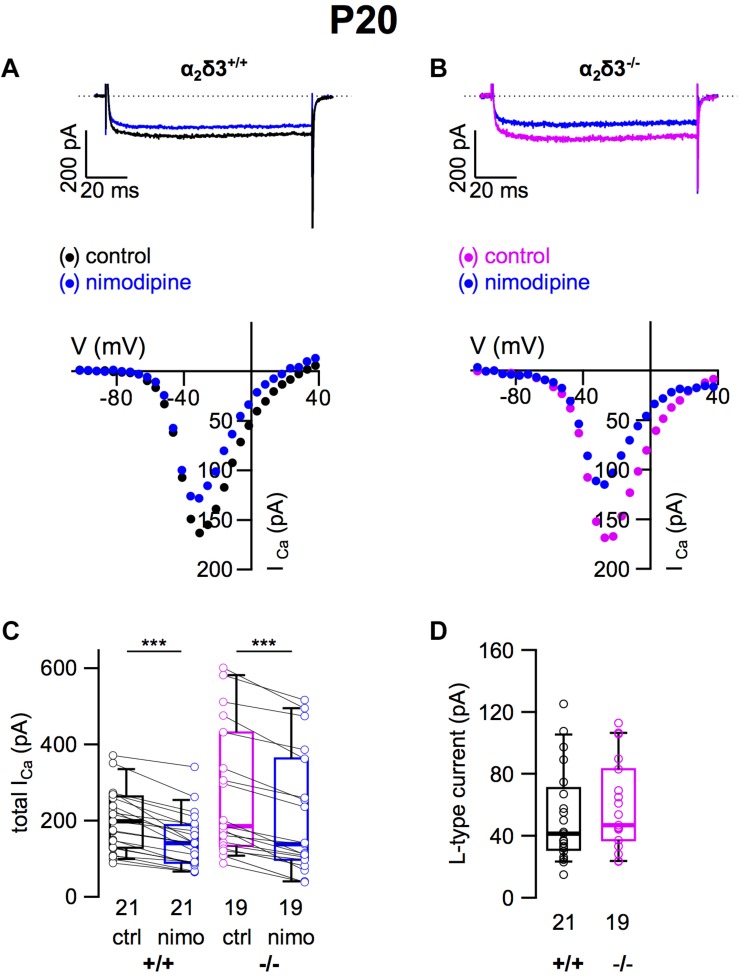
L-type Ca^2+^ currents are not altered in SG neurons of α_2_δ3^–/–^ mice at P20 + 3 DIV. **(A,B)** Maximum *I_Ca_* traces of an α_2_δ3^+/+^ (**A**, top) and an α_2_δ3^–/–^ SG neuron (**B**, top) in response to 100 ms depolarizing voltage steps before (α_2_δ3^+/+^, black; α_2_δ3^–/–^, magenta) and during application of 10 μM nimodipine (blue). Corresponding steady-state *I–V* curves are shown below the traces. **(C)** Box-and-whisker plots of *I_Ca_* (mean ± SD) before (ctrl) and under superfusion of 10 μM nimodipine (nimo) of SG neurons for α_2_δ3^+/+^ (+/+) and α_2_δ3^–/–^ (–/–) mice. Boxes represent the 25th–75th percentiles, the median (horizontal bar) and the 10th–90th percentiles (whiskers); paired individual data points are shown. Numbers under the box plots denote the numbers of SG neurons. Wilcoxon signed test, ^∗∗∗^*p* < 0.001. **(D)** Box-and-whisker plots with individual data points of the L-type current amplitude in SG neurons isolated from α_2_δ3^+/+^ (+/+) and α_2_δ3^–/–^ (–/–) mice. Wilcoxon rank test.

To assess the consequences of α_2_δ3 deletion for the expression of different types of voltage-gated Ca^2+^ channels, we recorded Ca^2+^ currents (*I_Ca_*) from primary cultured SG neurons that had been dissociated at P20 and cultured for 3 days. Cells with a large, round soma and one or two neurites were selected for patch-clamping, and there was no indication of an altered morphology of α_2_δ3^–/–^ SG neurons. L-type currents flowing through Ca_v_1.2 and Ca_v_1.3 channels, which both are expressed in mature SG neurons (Lv et al., 2014; Shrestha et al., 2018), were pharmacologically isolated using 10 μM nimodipine (Figures 1A,B). Control *I_Ca_* values before application of the blocker were quite variable in both genotypes shown by box plots and single data points (Figure 1C). Superfusion of 10 μM nimodipine significantly reduced the steady state *I_Ca_* in SG neurons in both α_2_δ3^+/+^ and α_2_δ3^–/–^ (Figure 1C and Table 1). The amplitude of L-type currents calculated as the differences between the respective control currents and the currents under superfusion with nimodipine was not different between genotypes (α_2_δ3^+/+^: 52.5 ± 30.4 pA, *n* = 21; α_2_δ3^–/–^: 59.0 ± 28.5 pA, *n* = 19; *p* = 0.17; Figure 1D). To summarize, lack of α_2_δ3 did not affect the average L-type current amplitude of 3-week-old primary cultured SG neurons compared with wildtype.

**TABLE 1:** Average steady-state Ca^2+^ current amplitudes (*I_Ca_*) before (control) and under superfusion with the respective Ca^2+^ current blocker/toxin (drug) ± SD for the isolation of different Ca^2+^ currents subtypes of SG neurons isolated at P20 from α_2_δ3^+/+^ and α_2_δ3^–/–^ mice.

**P20**	**α_2_δ3^+/+^**				**α_2_δ3^–/–^**			
**Isolation of/(drug)**	**control *I_Ca_* (pA)**	***I_Ca_* + drug (pA)**	**n**	***p***	**control *I_Ca_* (pA)**	***I_Ca_* + drug (pA)**	**n**	***p***
L-type (nimodipine)	200.0 ± 80.6	147.5 ± 71.0	21	<10^–6^	271.4 ± 169.9	212.5 ± 160.0	19	<10^–5^
P/Q-type (ω-agatoxin)	212.6 ± 74.8	97.3 ± 35.3	23	<10^–6^	221.8 ± 88.3	175.9 ± 74.3	27	<10^–7^
N-type (ω-conotoxin)	384.0 ± 127.8	353.1 ± 112.6	18	<10^–5^	272.6 ± 99.4	242.3 ± 94.1	15	<10^–4^
R-type (SNX 482)	373.9 ± 78.3	343.1 ± 68.0	13	<0.001	288.3 ± 82.8	269.2 ± 85.2	18	0.018
T-type (mibefradil)	387.7 ± 197.2	316.7 ± 160.0	19	<10^–5^	247.5 ± 111.0	153.6 ± 66.5	20	<10^–5^

*Number of SG neurons (n); *p* was determined using the Wilcoxon signed test.*

### P/Q-Type Ca^2+^ Currents Were Strongly Reduced in Cultured SG Neurons of 3-Week-Old α_2_δ3^–/–^ Mice

**FIGURE 2:**
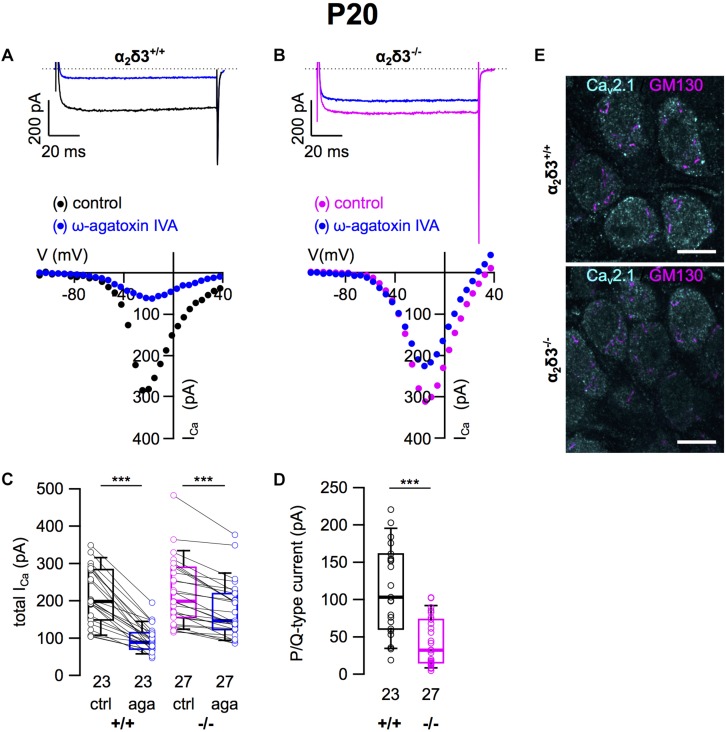
P/Q-type Ca^2+^ currents are strongly reduced in SG neurons of α_2_δ3^–/–^ mice at P20 + 3 DIV. **(A,B)** Maximum *I_Ca_* traces of an α_2_δ3^+/+^ (**A**, top) and an α_2_δ3^–/–^ SG neuron (**B**, top) in response to 100 ms depolarizing voltage steps before (α_2_δ3^+/+^, black; α_2_δ3^–/–^, magenta) and during application of 1 μM ω-agatoxin IVA (blue). Corresponding steady-state *I*–*V* curves are shown below the traces. **(C)** Box-and-whisker plots of *I_Ca_* before and during application of 1 μM ω-agatoxin IVA (aga) of SG neurons from α_2_δ3^+/+^ (+/+) and α_2_δ3^–/–^ (–/–) mice. Numbers under the box plots denote the numbers of SG neurons. Wilcoxon signed test, ^∗∗∗^*p* < 0.001. **(D)** Box-and-whisker plots showed a significant reduction of P/Q-type currents in SG neurons of α_2_δ3^–/–^ compared with α_2_δ3^+/+^ mice, Wilcoxon rank test, ^∗∗∗^*p* < 0.001. **(E)** Double immunolabeling of acutely dissected SG for Ca_v_2.1 and the Golgi marker GM130 in α_2_δ3^+/+^ (top) and α_2_δ3^–/–^ mice (bottom) at P20. Confocal images (single optical slices); 63×/1.4 NA oil objective; scale bar, 10 μm.

Next we analyzed whether lack of α_2_δ3 affected Ca_v_2.1 (P/Q) currents in cultured neurons at P20 (+ 3 DIV) using the P/Q-type channel blocker ω-agatoxin IVA (Figure 2). Superfusion of 1 μM ω-agatoxin IVA significantly reduced the steady state *I_Ca_* in SG neurons of α_2_δ3^+/+^ mice (Figures 2A,C and Table 1). A weaker but still significant block of *I_Ca_* was exerted by ω-agatoxin IVA in SG neurons of α_2_δ3^–/–^ mice (Figures 2B,C and Table 1). This indicates that deletion of α_2_δ3 strongly reduced the amplitude of P/Q-type currents from 115.3 ± 58.8 pA (*n* = 23) in wildtype to 45.9 ± 32.0 pA (*n* = 27; *p* < 10^–6^) in SG neurons from α_2_δ3^–/–^ mice (Figure 2D). In other words, P/Q-type currents comprise 54% of total *I_Ca_* in SG neurons of α_2_δ3^+/+^ mice. Lack of α_2_δ3 reduced P/Q-type currents by as much as 60.2% compared with α_2_δ3^+/+^ animals (Figure 2D).

Because α_2_δ proteins have been shown to participate in forward trafficking of Ca_v_2 channels to the plasma membrane and their final presynaptic destination (Kadurin et al., 2016; Dolphin, 2018), we tested for a potential aberrant intracellular localization of Ca_v_2.1 channels in α_2_δ3^–/–^ SG neurons by double immunolabeling acutely dissected SG tissue from 3-week-old mice for Ca_v_2.1 protein and the Golgi marker GM130 (Figure 2E). SG neurons showed both punctate and diffuse intracellular labeling and distinct punctate labeling for Ca_v_2.1 at the plasma membrane, which was weaker in α_2_δ3^–/–^ SG neurons compared with α_2_δ3^+/+^ and correlates with their smaller P/Q-type currents in cultured SG neurons. There was minor overlap of Ca_v_2.1 labeling with GM130 in both genotypes (Figure 2E), suggesting that the strongly reduced currents of α_2_δ3^–/–^ SG neurons were not caused by retention of Ca_v_2.1 channels within the cell, specifically in the Golgi apparatus.

### N-Type Ca^2+^ Currents Were Unaltered in Cultured SG Neurons of 3-Week-Old α_2_δ3^–/–^ Mice

**FIGURE 3:**
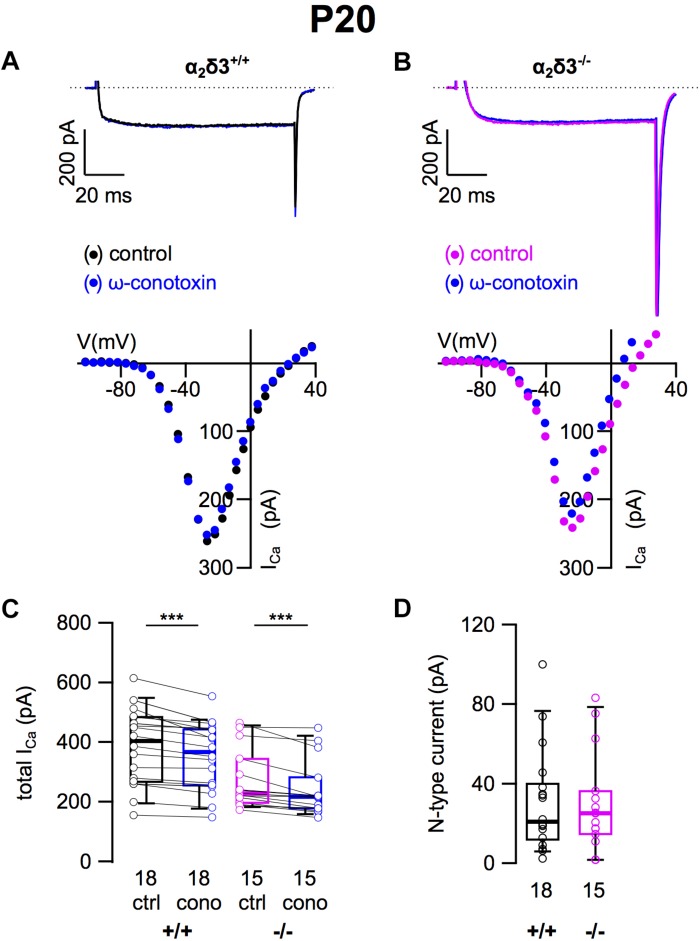
N-type Ca^2+^ currents are not altered in SG neurons of α_2_δ3^–/–^ mice at P20 + 3 DIV. **(A,B)** Maximum *I_Ca_* traces of an α_2_δ3^+/+^ (**A**, top) and an α_2_δ3^–/–^ SG neuron (**B**, top) in response to 100 ms depolarizing voltage steps before (α_2_δ3^+/+^, black; α_2_δ3^–/–^, magenta) and during application of 1 μM ω-conotoxin (blue). Corresponding steady-state *I*–*V* curves are shown below the traces. **(C)** Box-and-whisker plots of *I_Ca_* (mean ± SD) before (ctrl) and under superfusion of 1 μM ω-conotoxin (cono) of SG neurons from α_2_δ3^+/+^ (+/+) and α_2_δ3^–/–^ (–/–) mice. The number of neurons is indicated below the box plots. Wilcoxon signed test, ^∗∗∗^*p* < 0.001. **(D)** Box-and-whisker plots of N-type current amplitudes in SG neurons isolated from α_2_δ3^+/+^ and α_2_δ3^–/–^ mice. Wilcoxon rank test.

Because N-type and R-type currents have been recorded in SG neurons cultured from 3-month-old mice (Lv et al., 2012) we tested for the presence of these Ca_v_2 currents in α_2_δ3^–/–^ SG neurons. N-type currents were pharmacologically isolated using 1 μM ω-conotoxin (Figures 3A,B). For unknown reasons, the endogenous *I_Ca_* recorded from wildtype SG neurons was unusually large here (384.0 ± 127.8 pA, Table 1) and in recordings for isolation of R-type currents (see below) compared with values of SG neurons used to isolate L-type and P/Q-type currents (Figures 2C, 3C; 200.0 and 198.4 pA, respectively). We assume that this reflects the rather high variability of SG neurons in our culture.

Application of ω-conotoxin (1 μM) caused reductions of *I_Ca_* in α_2_δ3^+/+^ SG and α_2_δ3^–/–^ SG neurons (Figure 3C and Table 1). The amplitudes of the N-type currents were small (α_2_δ3^+/+^: 30.8 ± 25.8 pA, *n* = 18; α_2_δ3^–/–^: 30.2 ± 24.8 pA, *n* = 15) and were not different between α_2_δ3^+/+^ and α_2_δ3^–/–^ mice (*p* = 0.47; Figure 3D). These data indicate that lack of α_2_δ3 did not affect the small N-type currents in SG neurons of our preparation. Further, N-type currents did not compensate for the loss of P/Q-type channels in α_2_δ3^–/–^ mice.

### Small R-Type Ca^2+^ Currents Were Reduced in Cultured SG Neurons of 3-Week-Old α_2_δ3^–/–^ Mice

**FIGURE 4:**
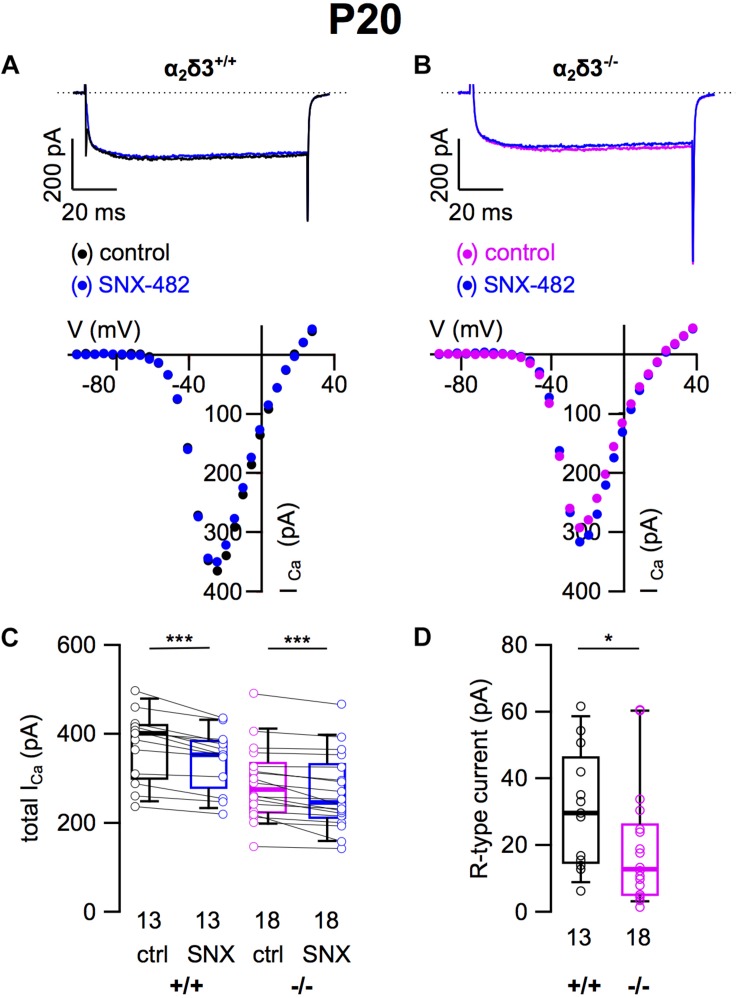
Small R-type Ca^2+^ currents are reduced in SG neurons of α_2_δ3^–/–^ mice at P20 + 3 DIV. **(A,B)** Maximum *I_Ca_* traces of an α_2_δ3^+/+^ (**A**, top) and an α_2_δ3^–/–^ SG neuron (**B**, top) in response to 100 ms depolarizing voltage steps before (α_2_δ3^+/+^, black; α_2_δ3^–/–^, magenta) and during application of 1 μM SNX-482 (blue). Corresponding steady-state *I*–*V* curves are shown below the traces. **(C)** Box-and-whisker plots of *I_Ca_* before (ctrl) and under superfusion of 1 μM SNX-482 of SG neurons isolated from α_2_δ3^+/+^ (+/+) and α_2_δ3^–/–^ (–/–) mice. The number of neurons is indicated below the box plots. Wilcoxon signed test, ^∗∗∗^*p* < 0.001. **(D)** Box-and-whisker plots showed a significant reduction of R-type current amplitudes in SG neurons from α_2_δ3^–/–^ compared with α_2_δ3^+/+^ mice. Wilcoxon rank test, ^*^p < 0.05.

To assess the contribution of R-type (Ca_v_2.3) currents to total *I_Ca_* of SG neurons and to test whether lack of α_2_δ3 affected their expression, we employed the Ca_v_2.3 channel blocker SNX 482 (Figures 4A,B). SNX 482 (1 μM) significantly reduced *I_Ca_* in α_2_δ3^+/+^ and α_2_δ3^–/–^ SG neurons (Figure 4C and Table 1). The small R-type currents of α_2_δ3^+/+^ (30.8 ± 17.6 pA, *n* = 13) were further reduced in α_2_δ3^–/–^ SG neurons (19.1 ± 17.7 pA, *n* = 18; *p* = 0.018; Figure 4D). To summarize, deletion of α_2_δ3 reduced the small amplitude of R-type currents in SG neurons by 38%, and R-type currents did not compensate for the reduction of P/Q-type currents in α_2_δ3^–/–^ mice.

### T-Type Ca^2+^ Currents Were Unaltered in Cultured SG Neurons of 3-Week-Old α_2_δ3^–/–^ Mice

**FIGURE 5:**
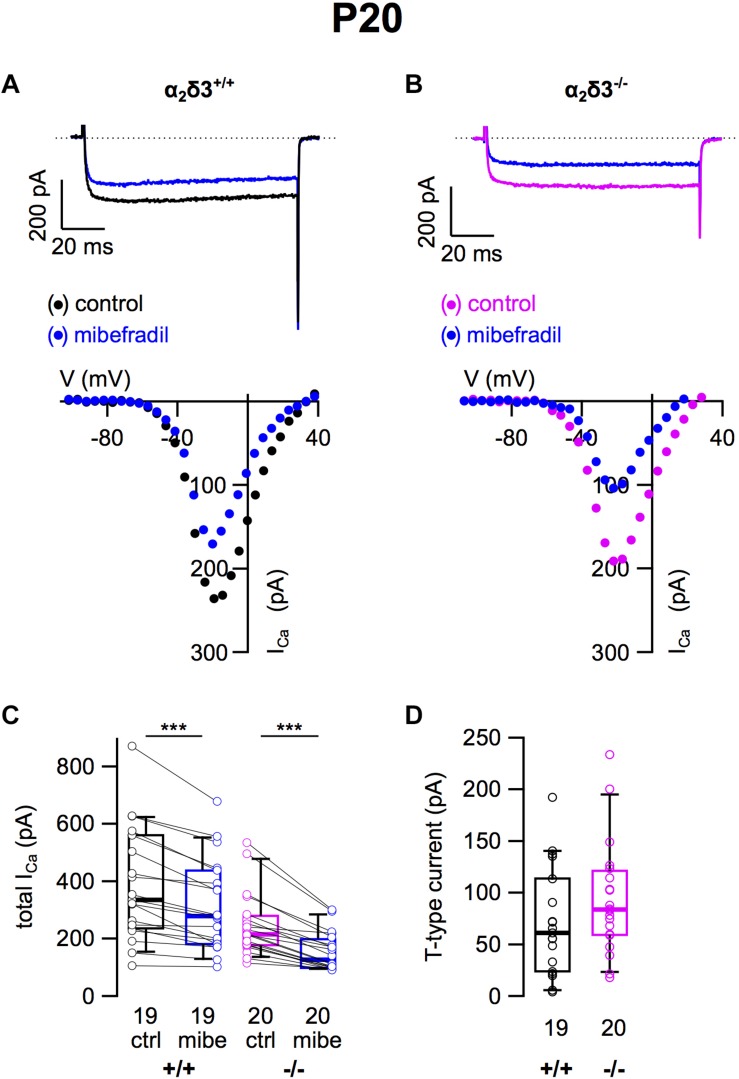
T-type Ca^2+^ currents are not altered in SG neurons of α_2_δ3^–/–^ mice at P20 + 3 DIV. **(A,B)** Maximum *I_Ca_* traces of an α_2_δ3^+/+^ (**A**, top) and an α_2_δ3^–/–^ SG neuron (**B**, top) in response to 100 ms depolarizing voltage steps before (α_2_δ3^+/+^, black; α_2_δ3^–/–^, magenta) and during application of 5 μM mibefradil (blue). Corresponding steady-state *I*–*V* curves are shown below the traces. **(C)** Box-and-whisker plots of *I_Ca_* before (ctrl) and under superfusion of 5 μM mibefradil (mibe) of SG neurons isolated from α_2_δ3^+/+^ (+/+) and α_2_δ3^–/–^ mice (–/–). The number of neurons is indicated below the box plots. Wilcoxon signed test, ^∗∗∗^*p* < 0.001. **(D)** Box-and-whisker plots of highly variable T-type current amplitudes in SG neurons isolated from α_2_δ3^+/+^ and α_2_δ3^–/–^ mice. Wilcoxon rank test.

Finally, we tested for T-type (Ca_v_3) currents, which activate at more negative potentials than Ca_v_1 and Ca_v_2 channels and do neither require α_2_δ nor β subunits for their membrane expression (Dolphin, 2013). They were pharmacologically isolated using 5 μM mibefradil (Lv et al., 2012; Figure 5). The example *I–V* curves before/under mibefradil indicate a larger difference (T-type) component in the α_2_δ3^–/–^ neuron of ∼100 pA (Figure 5B) compared with the α_2_δ3^+/+^ neuron (Figure 5A). Under superfusion with mibefradil, *I_Ca_* of the α_2_δ3^–/–^ SG neuron indeed activated 5–10 mV more positive than before indicating that a substantial, negatively activating *I_Ca_* (T-type) component was blocked (Figure 5B). On average, application of mibefradil reduced the steady state *I_Ca_* in α_2_δ3^+/+^ and in α_2_δ3^–/–^ SG neurons (Figure 5C and Table 1). Isolated T-type currents were highly variable between neurons of either genotype (Figure 5D). There was a tendency of increased T-type currents in α_2_δ3^–/–^ SG neurons, which, however, was not significant (α_2_δ3^+/+^: 71.0 ± 52.1 pA, *n* = 19; α_2_δ3^–/–^: 94.2 ± 56.0 pA, *n* = 20; *p* = 0.081), most likely because of the large scatter in T-type currents (Figure 5D).

### Total Ca^2+^ Currents and Amplitudes of Ca^2+^ Current Subtypes in Cultured SG Neurons of 3-Week-Old α_2_δ3^+/+^ and α_2_δ3^–/–^ Mice

**FIGURE 6:**
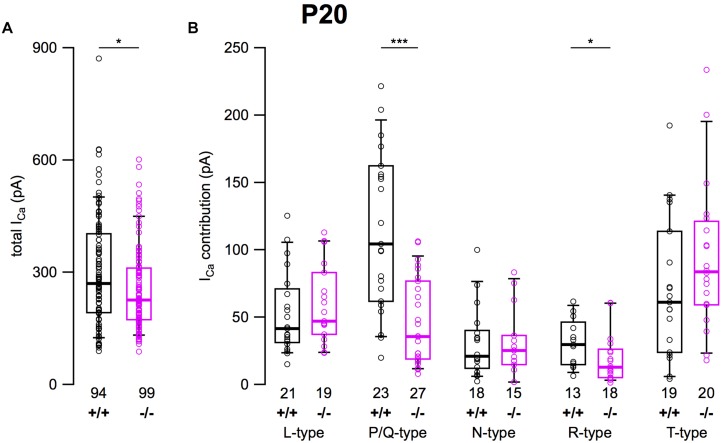
Effects of deletion of α_2_δ3 on total *I_Ca_* and *I_Ca_* components of SG neurons isolated at P20 + 3 DIV. **(A)** Box-and-whisker plots indicated that total *I_Ca_* of SG neurons was smaller in α_2_δ3^–/–^ (–/–) versus α_2_δ3^+/+^ (+/+) mice. **(B)** Box-and-whisker plots of the amplitudes of different voltage-activated Ca^2+^ current types of SG neurons from α_2_δ3^+/+^ (+/+) and α_2_δ3^–/–^ (–/–) mice. Both the dominant P/Q-type currents as well as the much smaller R-type currents of α_2_δ3^+/+^ mice were reduced in α_2_δ3^–/–^ mice. The number of neurons is indicated below the boxes. Wilcoxon rank test, ^*^p < 0.05, ^∗∗∗^*p* < 0.001.

After determining the amplitudes of *I_Ca_* in SG neurons mediated by L-, P/Q-, N-, R-, and T-type currents, we summarized their amplitudes and the total current in SG neurons of α_2_δ3^+/+^ and α_2_δ3^–/–^ mice (Figure 6). The average total *I_Ca_* of SG neurons dissociated at P20 (+ 3 DIV) was significantly smaller in α_2_δ3^–/–^ mice (256.3 ± 113.9 pA, *n* = 99) corresponding to 85.3% of α_2_δ3^+/+^ (300.3 ± 147.9 pA, *n* = 94; *p* = 0.019; Figure 6A). In wildtype SG neurons, P/Q-type currents were the dominant Ca^2+^ current component (115.3 ± 58.8 pA), which was reduced to 45.9 ± 32.0 pA in α_2_δ3^–/–^ (or 39.8% of the wildtype value, Figure 6B). Small R-type currents of α_2_δ3^+/+^ SG neurons (30.8 ± 17.6 pA) were significantly reduced to 19.1 ± 17.7 pA in α_2_δ3^–/–^ SG neurons. T-type currents were the second largest *I_Ca_* component (71.0 ± 52.1 pA) in α_2_δ3^+/+^ SG neurons at P20 (+ 3 DIV), and there was a tendency of increased T-type currents in α_2_δ3^–/–^ mice (94.2 ± 56.0 pA, Figure 6B). Notably, T-type (Ca_v_3) currents comprise a VGCC family that does not co-assemble with an auxiliary α_2_δ subunit. Neither L- nor N-type currents compensated for the loss of P/Q currents in α_2_δ3^–/–^ SG neurons. These data indicate that upon lack of α_2_δ3 (i) Ca_v_2.1 and Ca_v_2.3 current sizes of SG neurons could not be fully compensated by the isoforms α_2_δ1 or α_2_δ2 and (ii) partial compensation of total *I_Ca_* did not rely on up-regulation of Ca_v_1 and Ca_v_2 family members.

Patch-clamp recordings from wildtype endbulbs in slices of the anteroventral cochlear nucleus of P9–P13 old mice with local pharmacological block revealed 85% P/Q- and 15% N-type presynaptic Ca^2+^ currents (Lin et al., 2011). Although the presynaptic complement of Ca^2+^ channels is different from that at the soma (Doughty et al., 1998) and we moreover used primary cultured SG neurons, the requirement for α_2_δ3 to yield normal P/Q currents in those neurons may suggest a reduced number of presynaptic P/Q channels at the endbulb synapse in 3-week-old mice α_2_δ3^–/–^ mice. The question arises as to the role of α_2_δ3 on the composition of *I_Ca_* well before the onset of hearing. We therefore analyzed a subset of *I_Ca_* types in neonatal SG neurons dissociated at P5 and cultured for 2 DIV.

### L-Type Ca^2+^ Currents Were Reduced in Cultured SG Neurons of Neonatal α_2_δ3^–/–^ Mice

**FIGURE 7:**
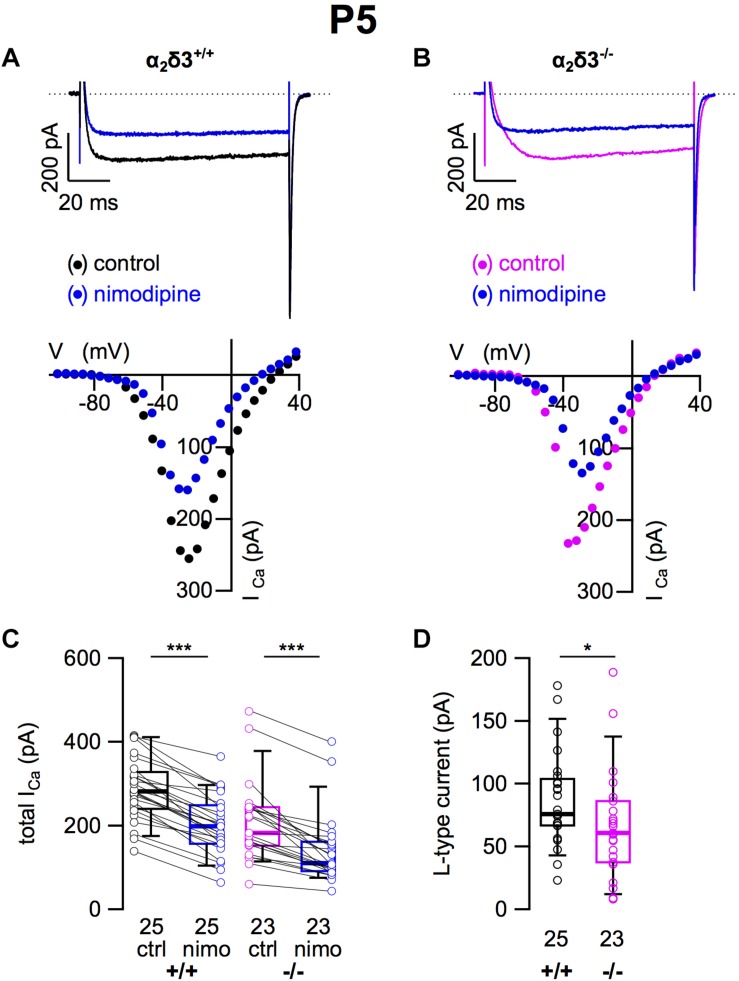
L-type Ca^2+^ currents are reduced in neonatal SG neurons of α_2_δ3^–/–^ mice at P5 + 2 DIV. **(A,B)** Maximum *I_Ca_* traces of an α_2_δ3^+/+^ (**A**, top) and an α_2_δ3^–/–^ SG neuron (**B**, top) in response to 100 ms depolarizing voltage steps before (α_2_δ3^+/+^, black; α_2_δ3^–/–^, magenta) and during application of 10 μM nimodipine (blue). Corresponding steady-state *I*–*V* curves are shown below the traces. **(C)** Box-and-whisker plots of *I_Ca_* before (ctrl) and under superfusion of 10 μM nimodipine (nimo) of SG neurons isolated from α_2_δ3^+/+^ (+/+) and α_2_δ3^–/–^ (–/–) mice. Numbers under the box plots denote the numbers of SG neurons. Wilcoxon signed test, ^∗∗∗^*p* < 0.001. **(D)** Box plots of the L-type current amplitude show a reduction in SG neurons from α_2_δ3^–/–^ compared with wildtype mice; Wilcoxon rank test, ^*^p < 0.05.

Because L-type currents mediated by Ca_v_1.2 and Ca_v_1.3 channels play a role in Ca^2+^ signals controlling gene transcription, neurite growth, and neuronal differentiation (Dolmetsch et al., 2001; Roehm et al., 2008; Satheesh et al., 2012), we recorded those currents in SG neurons cultured at P5 (+ 2 DIV). SG neurons isolated from α_2_δ3^–/–^ mice did not show an immature or altered morphology compared with those of wildtype. L-type currents were present in SG neurons of both α_2_δ3^+/+^ and α_2_δ3^–/–^ mice (Figures 7A,B). Nimodipine (10 μM) blocked part of the total steady-state current in SG neurons of α_2_δ3^+/+^ and α_2_δ3^–/–^ mice (Figure 7C and Table 2). Lack of α_2_δ3 reduced the L-type current amplitude to 66.7 ± 43.4 pA (*n* = 23) or 76.7% compared with α_2_δ3^+/+^ (87.0 ± 37.2 pA, *n* = 25; *p* = 0.017; Figure 7C). Notably, the average size of L-type currents in wildtype SG neurons was larger at P5 (87.0 ± 37.2 pA; Figure 7D) compared with P20 (52.5 ± 30.4 pA; *p* < 0.001; Figure 1D).

**TABLE 2:** Average steady-state Ca^2+^ current amplitudes (*I_Ca_*) before (control) and under superfusion with the respective Ca^2+^ current blocker/toxin (drug) ± SD for the isolation of different Ca^2+^ currents subtypes of SG neurons isolated at P5 from α_2_δ3^+/+^ and α_2_δ3^–/–^ mice.

**P5**	**α_2_δ3^+/+^**				**α_2_δ3**^–/–^			
**Isolation of/(drug)**	**control *I_Ca_* (pA)**	***I_Ca_* + drug (pA)**	**n**	***p***	**control *I_Ca_* (pA)**	***I_Ca_* + drug (pA)**	**n**	***p***
L-type (nimodipine)	287.5 ± 73.7	200.5 ± 71.2	25	<10^–7^	208.8 ± 95.9	142.1 ± 84.1	23	<10^–6^
P/Q-type (ω-agatoxin)	279.3 ± 85.4	235.5 ± 63.3	14	<10^–3^	282.3 ± 61.4	249.9 ± 77.4	14	<10^–3^
N-type (ω-conotoxin)	295.9 ± 82.3	202.0 ± 52.5	21	<10^–6^	278.3 ± 165.0	225.0 ± 166.8	17	<10^–4^

*Number of SG neurons (n); *p* was determined using the Wilcoxon signed test.*

At the age of P5, the average unblocked *I_Ca_* of all wildtype SG neurons amounted to 288.7 ± 78.5 pA (*n* = 60), which was reduced to 249.8 ± 119.3 pA (86.5%) in α_2_δ3^–/–^ SG neurons (*n* = 54; *p* = 0.006). These values are very similar to average total *I_Ca_* values of SG neurons isolated at P20 of 300.3 pA for α_2_δ3^+/+^ and of 256.3 pA for α_2_δ3^–/–^ mice (cf. Figure 6A). Whereas L-type currents in P20 SG neurons were not different between both genotypes (cf. Figure 1D), they were clearly smaller in SG neurons of α_2_δ3^–/–^ versus α_2_δ3^+/+^ mice isolated at P5.

### Small P/Q-Type Ca^2+^ Currents Were Not Altered in Cultured SG Neurons of Neonatal α_2_δ3^–/–^ Mice

**FIGURE 8:**
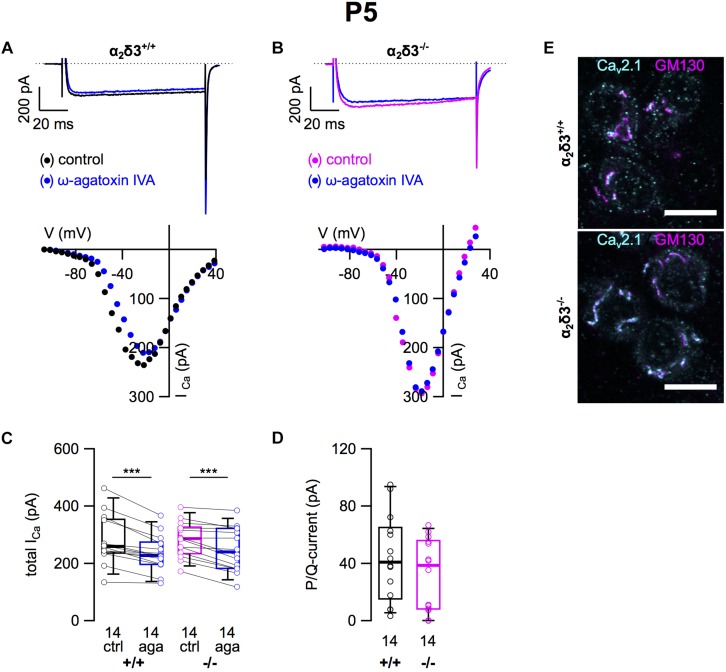
Small P/Q-type Ca^2+^ currents are not altered in neonatal SG neurons of α_2_δ3^–/–^ mice at P5 + 2 DIV. **(A,B)** Maximum *I_Ca_* traces of an α_2_δ3^+/+^ (**A**, top) and an α_2_δ3^–/–^ SG neuron (**B**, top) in response to 100 ms depolarizing voltage steps before (α_2_δ3^+/+^, black; α_2_δ3^–/–^, magenta) and during application of 1 μM ω-agatoxin IVA (blue). Corresponding steady-state *I*–*V* curves are shown below the traces. **(C)** Box-and-whisker plots of *I_Ca_* before (ctrl) and under superfusion of 1 μM ω-agatoxin IVA (aga) of SG neurons isolated from α_2_δ3^+/+^ (+/+) and α_2_δ3^–/–^ (–/–) mice. Numbers under the box plots denote the numbers of SG neurons. Wilcoxon signed test, ^∗∗∗^*p* < 0.001. **(D)** P/Q-type current amplitudes were unaltered between SG neurons from both genotypes; Wilcoxon rank test. **(E)** Double immunolabeling for Ca_v_2.1 (cyan) and the Golgi marker GM 130 (magenta) in acutely dissected SG tissue of α_2_δ3^+/+^ (top) and α_2_δ3^–/–^ (bottom) at P5. In both genotypes, most of the Ca_v_2.1 protein was localized in the Golgi apparatus (overlap of magenta and cyan, resulting in white color) rather than in the membrane of the three SG neurons each (weak dotted outline in cyan). Confocal images (single optical slices); 63×/1.4 NA oil objective; scale bar, 10 μm.

Next we analyzed P/Q-type currents in P5 (+ 2 DIV) SG neurons by using 1 μM ω-agatoxin IVA (Figures 8A,B). The decrease of *I_Ca_* by the blocker was significant for SG neurons of α_2_δ3^+/+^ and α_2_δ3^–/–^ mice (Figure 8C and Table 2). The difference currents indicate a heterogenous but on average small contribution of P/Q-type currents to total *I_Ca_* in both α_2_δ3^+/+^ (43.9 ± 29.9 pA, *n* = 14) and α_2_δ3^–/–^ mice (32.4 ± 25.0 pA, *n* = 14; Figure 8D). In contrast to our findings in P20 neurons, the P/Q-current amplitude was not different between the two genotypes (*p* = 0.17).

Double immunolabeling of acutely dissected SG tissue at P5 revealed sparse dot-like labeling of P/Q-type channels at the plasma membrane of SG neurons (Figure 8E). Most of the Ca_v_2.1 immunofluorescence was, however, co-localized with GM130 in the Golgi apparatus of both α_2_δ3^+/+^ and α_2_δ3^–/–^ neurons (Figure 8E), much in contrast to 3-week-old SG neurons (Figure 2E). To summarize, Ca_v_2.1 channels are just being up-regulated, synthesized, and inserted into the plasma membrane of SG neurons at P5 leading to similar small P/Q currents at this age, which was independent of the presence of the α_2_δ3 subunit. This raises the question whether α_2_δ3 is expressed in WT neurons at this age at all.

### Transcript Analysis of *Cacna2d* Isoforms in Spiral Ganglia of Neonatal Mice

**FIGURE 9:**
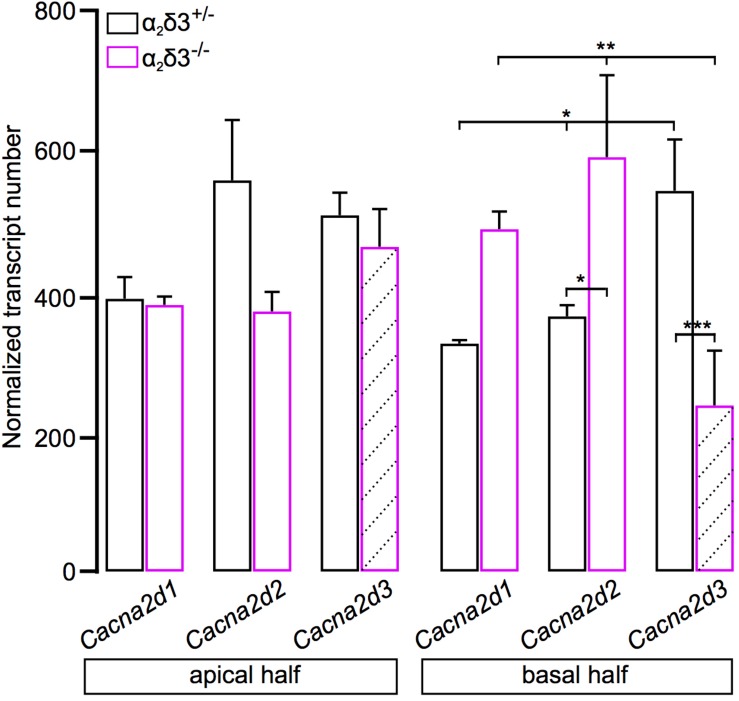
Transcript analysis for *Cacna2d1*, *Cacna2d2*, and *Cacna2d3* in spiral ganglion tissue of neonatal α_2_δ3^+/–^ and α_2_δ3^–/–^ mice (P5) using qRT-PCR. Transcript numbers (mean ± SEM) normalized to *Hprt1* and *Sdha* of cDNA synthesized from SG tissue from the apical (left) or the basal cochlea cochlear half (right). Note that due to the design of the α_2_δ3 knockout construct (introduction of a frameshift in exon 15, see section “Materials and Methods”) and the position of α_2_δ3-specific primers for qPCR (junction between exon 5 and exon 6), α_2_δ3-specific transcripts were still detected in α_2_δ3^–/–^ tissue (hatched bars). However, functional protein will not be produced. Each column is from 3 to 4 independent samples. Two-way ANOVA was performed on log10-transformed transcript numbers with Holm–Sidak *post hoc* test; ^*^p < 0.05, ^∗∗^*p* < 0.01, ^∗∗∗^*p* < 0.001.

Transcripts for *Cacna2d1*, *Cacna2d2*, and *Cacna2d3* encoding the three neuronal subunits α_2_δ1, α_2_δ2 and α_2_δ3 have been identified in SG neurons of 3-week-old mice (Shrestha et al., 2018) but it was unclear whether they are present in neonatal SG neurons. We performed transcript analysis for *Cacna2d1*, *Cacna2d2*, and *Cacna2d3* with quantitative RT-PCR using SG tissue of P5 α_2_δ3^–/–^ mice with α_2_δ3^+/–^ littermates serving as control. All three neuronal subunits *Cacna2d1*, *Cacna2d2*, and *Cacna2d3* were expressed in α_2_δ3^+/–^ SG tissue of the apical and basal cochlear halves (Figure 9). Note that due to the design of the α_2_δ3 knockout construct (introduction of a frameshift in exon 15) and the position of α_2_δ3-specific primers for qPCR (junction between exon 5 and exon 6), truncated, non-functional α_2_δ3-specific transcripts were still detected in α_2_δ3^–/–^ tissue (hatched bars in Figure 9; see section “Materials and Methods”). There was no difference in the total amount of *Cacna2d1–3* subunit mRNA between α_2_δ3^+/–^ and α_2_δ3^–/–^ cells (two-way ANOVA; apex, *F* = 4.11, *p* = 0.058; base, *F* = 0.03; *p* = 0.876). Transcript numbers of individual subunits were not different between the two genotypes in the apical half (*F* = 2.12; *p* = 0.149). However, in α_2_δ3^+/–^ neurons of the basal half, *Cacna2d3* expression was significantly higher than expression of *Cacna2d1* (*p* = 0.03) but not significantly higher than *Cacna2d2* (*p* = 0.085). Expression of *Cacna2d3* was significantly lower in α_2_δ3^–/–^ compared with α_2_δ3^+/–^ cells (*p* < 0.001). There was a tendency toward increased levels of both *Cacna2d1* and *Cacna2d2* in α_2_δ3^–/–^ cells but these differences were not significant (Figure 9). To summarize, *Cacna2d1* and *Cacna2d2* transcripts partially compensate for non-functional *Cacna2d3* transcripts in the knockout. Importantly, *Cacna2d3* mRNA is present in α_2_δ3^+/–^ SG neurons in the entire cochlea at P5, suggesting that the small amplitude of P/Q currents at this age was not caused by the developmental absence of α_2_δ3. Rather, the similar small amplitudes of P/Q currents in both α_2_δ3^+/+^ and α_2_δ3^–/–^ SG neurons (Figure 8D) infer that they do not depend on the presence of the α_2_δ3 subunit at this age.

### N-Type Ca^2+^ Currents Were Severely Reduced in Cultured SG Neurons of Neonatal α_2_δ3^–/–^ Mice

**FIGURE 10:**
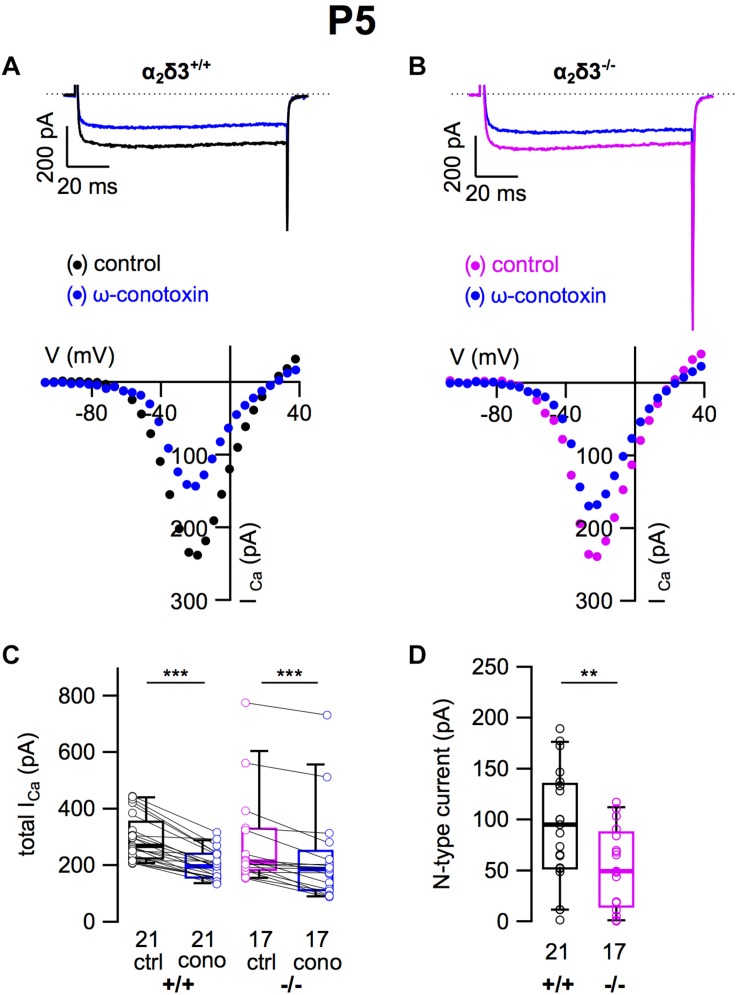
N-type Ca^2+^ currents are substantially reduced in neonatal SG neurons of α_2_δ3^–/–^ mice at P5 + 2 DIV. **(A,B)** Maximum *I_Ca_* traces of an α_2_δ3^+/+^ (**A**, top) and an α_2_δ3^–/–^ SG neuron (**B**, top) in response to 100 ms depolarizing voltage steps before (α_2_δ3^+/+^, black; α_2_δ3^–/–^, magenta) and during application of 1 μM ω-conotoxin (blue). Corresponding steady-state *I*–*V* curves are shown below the traces. **(C)** Box-and-whisker plots of *I_Ca_* before (ctrl) and under superfusion of 1 μM ω-conotoxin (cono) of SG neurons isolated from α_2_δ3^+/+^ (+/+) and α_2_δ3^–/–^ mice (–/–). Numbers under the boxes denote the numbers of SG neurons. Wilcoxon signed test, ^∗∗∗^*p* < 0.001. **(D)** N-type current amplitudes of SG neurons were variable in both genotypes but were significantly reduced in SG neurons of α_2_δ3^–/–^ mice. Wilcoxon rank test, ^∗∗^*p* < 0.01.

A switch of N-type to P/Q-type currents has been reported in early postnatal development of the calyx of Held synapse, inhibitory thalamic and cerebellar synapses and the neuromuscular junction (Siri and Uchitel, 1999; Iwasaki et al., 2000). Therefore we tested whether neonatal SG neurons express N-type currents and if they were affected by deletion of α_2_δ3 (Figures 10A,B). Application of ω-conotoxin (1 μM) caused significant reductions in *I_Ca_* in α_2_δ3^+/+^ SG neurons (Figure 10C and Table 2). In SG neurons of α_2_δ3^–/–^ mice, there was a weaker response to ω-conotoxin (Figure 10C and Table 2). Deletion of α_2_δ3 significantly reduced the amplitude of N-type currents in α_2_δ3^+/+^ SG neurons from 94.4 ± 55.2 pA (*n* = 21) to 53.3 ± 36.5 pA (*n* = 17; *p* = 0.009) in α_2_δ3^–/–^ SG neurons (Figure 10D). Taken together, cultured SG neurons of wildtype mice at P5 + 2 DIV displayed N-type currents that were 3.1 times larger as at P20 + 3 DIV (30.8 pA, cf. Figure 3D). N-type currents of neonatal SG neurons deficient for α_2_δ3 were reduced to 56% of the wildtype value, suggesting that α_2_δ3 is indispensible for normal expression of Ca_v_2.2 channels at this age.

## Discussion

In this study, we have analyzed the role of the auxiliary Ca^2+^ channel subunit α_2_δ3 for the size and composition of Ca^2+^ currents of dissociated primary cultured SG neurons in the mature (P20) and immature cochlea (P5). The impaired *in vivo* function of the endbulb of Held synapse in α_2_δ3^–/–^ mice (Pirone et al., 2014) led us to analyze whether lack of α_2_δ3 specifically affected voltage-activated Ca^2+^ currents. Due to the small size of endbulb synapses in α_2_δ3^–/–^ mice, recordings of presynaptic currents are not feasible. Though the composition of Ca^2+^ currents differs between the presynapse and the soma (Doughty et al., 1998) our approach can nevertheless give insights into the specific dependence of somatic Ca^2+^ currents on the expression of α_2_δ3, which ultimately may also affect presynaptic (Ca_v_2.1) currents (Lübbert et al., 2019).

### Composition of Ca^2+^ Currents in SG Neurons of 3-Week-Old Mice

SG neurons isolated from wildtype mice at P20 expressed different voltage-activated Ca^2+^ channels that conducted L-type, P/Q-type, N-type, R-type, and T-type currents. P/Q currents carried by Ca_v_2.1 channels formed the largest *I_Ca_* component comprising 54% of the total *I_Ca_*, which contrasts findings from Lv et al. (2012) who found 11.5% P/Q current for basal and 17.2% for apical SG neurons in 3-month-old mice. However, the authors used a different subpopulation of neurons by separately culturing the most apical and the most basal part of the SG while discarding the middle part, which represents the range of best hearing. In contrast, our SG neurons were cultured from the apical and the basal half of the cochlea, respectively, such that SG neurons from the middle region were included in both cultures. The different age (3 weeks versus 3 months) may also be a factor, potentially leading to reduced Ca_v_2.1 currents in the fully mature subpopulation of SG neurons used by Lv et al. (2012). In line with our *I_Ca_* data, recent single cell RNA sequencing demonstrated strong *Cacna1a* expression (encoding for Ca_v_2.1) in 4-week-old mice, which dominated all transcripts for VGCCs in type I SG neurons (Shrestha et al., 2018).

An alternative explanation for the discrepancy in P/Q currents in SG neurons of wildtype mice, cf. (Lv et al., 2012), may lie in the dissociated primary culture of SG itself, which results in rather low neuronal survival (Vieira et al., 2007). Moreover, because type I SG neurons forming subclasses Ia, Ib, and Ic are molecularly and physiologically heterogeneous (Petitpré et al., 2018; Shrestha et al., 2018; Sun et al., 2018), subtle differences in dissociation protocols or chemicals may have resulted in a differential survival of these subclasses (Liu et al., 2016; Browne et al., 2017; Cai et al., 2017). Differential survival of subclasses may also explain the heterogeneity in our *I_Ca_* values for a given genotype and age.

### Lack of α_2_δ3 Severely Affects Ca_v_2.1 Currents in Cultured SG Neurons of 3-Week-Old Mice

The reduction of P/Q currents of α_2_δ3^–/–^ SG neurons by 58% compared with the wildtype (Figures 2, 6) is in line with a significant reduction of immunopositive Ca_v_2.1 puncta at the plasma membrane and within the somata of α_2_δ3^–/–^ SG neurons (cf. Figure 2E). Reduction of somatic P/Q channels may lead to decreased axonal trafficking and channel density at the presynaptic terminal *in vivo* (Lin et al., 2011; Kadurin et al., 2016) and might contribute to the impaired function of the endbulb of Held synapse in α_2_δ3^–/–^ mice (Pirone et al., 2014). In this context, a recent study showed that specific overexpression of Ca_v_2.1 channels increased presynaptic currents and synaptic strength of the calyx of Held synapse, indicating that the somatic expression of Ca_v_2.1 regulated the presynaptic abundance of Ca_v_2.1 channels at this synapse (Lübbert et al., 2019).

Our results of reduced somatic P/Q currents in α_2_δ3^–/–^ SG neurons contrast recent findings of Landmann et al. (2018) who reported unaltered expression of Ca_v_2.1 channels accompanied by increased expression of Ca_v_2.2 and Ca_v_2.3 protein in the primary somatosensory and motor cortex of α_2_δ3^–/–^ mice. However, despite up-regulation of both the number of Ca_v_2.2- and Ca_v_2.3-positive neurons and of Ca_v_2.2 and Ca_v_2.3 channels at the subcellular level the thermal nociceptive pathway failed in α_2_δ3^–/–^ mice (Landmann et al., 2018) suggesting that VGCC-independent functions of α_2_δ3 played a role (Dolphin, 2018).

In cerebellar Purkinje cells, Ca_v_2.1 channels require the α_2_δ2 subunit for proper cellular function and normal morphology of the dendritic tree as shown for an α_2_δ2 null mutant, the ducky mouse (Brodbeck et al., 2002). Such a phenotype has not been found in α_2_δ3^–/–^ mice (Neely et al., 2010) further corroborating the view that a specific pairing of a particular Ca_v_ channel type with a defined α_2_δ isoform does not exist in general. Co-assembly between the two partners rather depends on the cellular and extracellular context (Fell et al., 2016; Dolphin, 2018).

Not only P/Q-type currents, but also R-type currents were significantly affected by deletion of α_2_δ3. Mean R-type currents, which amounted to only 31 pA in wildtype SG neurons, were further reduced to 19.1 pA (or 62%) in α_2_δ3^–/–^ SG neurons. The total *I_Ca_* of SG neurons in our preparation was reduced in α_2_δ3^–/–^ SG neurons to 85% of the wildtype yet to a lesser extent than the reduction in P/Q- and R-type currents should have inferred. A similar result has been found for total *I_Ca_* in cultured DRG neurons from α_2_δ3^–/–^ mice (Neely et al., 2010). The question arises as to the nature of the compensatory *I_Ca_* component. T-type currents, which are present in SG neurons (Lv et al., 2012), are likely candidates to compensate for the reduction in P/Q currents because they can form functional channels without co-assembling with any α_2_δ subunit, for review see Dolphin (2012, 2018). Indeed, there was a tendency of increased T-type currents in α_2_δ3^–/–^ SG neurons (Figures 5, 6). Notably, T-type currents were increased in thalamocortical relay neurons of a mouse line with loss-of-function of Ca_v_2.1 (Zhang et al., 2002), which is partially recapitulated by the severe reduction of P/Q (Ca_v_2.1) currents in P20 SG neurons of α_2_δ3^–/–^ mice in the present study.

### Opposing Effects of Deletion of α_2_δ3 on L-Type, N-Type, and P/Q-Type Currents in Cultured Neonatal SG Neurons

So far, voltage-activated Ca^2+^ currents have not been analyzed in neonatal SG neurons. Our recordings of L-, N-, and P/Q-type currents from P5 wildtype SG neurons revealed amplitudes of the *I_Ca_* components that clearly differed from those at P20. At P5, L-type currents were 1.67-fold larger and N-type currents even 3.1-fold larger compared with P20. Moreover, deletion of α_2_δ3 significantly reduced L-type and N-type currents in P5 SG neurons whereas it had no effect on L-type and N-type currents at P20.

Neonatal SG neurons (P5 + 2 DIV) of α_2_δ3^+/+^ and α_2_δ3^–/–^ mice exhibited similar small P/Q currents. This finding was supported by sparse Ca_v_2.1 immunoreactivity in the somatic membrane whereas it was strongly enriched in the Golgi apparatus of SG neurons at P5 suggesting that Ca_v_2.1 channels were just being up-regulated in both α_2_δ3 wildtype and knockout (Figure 8). At P20, P/Q currents were 2.6-fold larger than at P5 and comprised the largest *I_Ca_* component of cultured α_2_δ3^+/+^ SG neurons. In contrast, P/Q currents of α_2_δ3^–/–^ SG neurons at P20 had risen only 1.4-fold.

Immature SG neurons relay information from spontaneously active IHCs in a burst-firing mode with maximum firing rates of 100–300 Hz until the end of the first postnatal week (Tritsch and Bergles, 2010; Tritsch et al., 2010). With the onset of hearing, IHCs produce sound-evoked receptor potentials leading to much higher firing rates in type I SG neurons (Taberner and Liberman, 2005; Wu et al., 2016), which are accompanied by differentiation into SG neuron subtypes Ia, Ib, and Ic and respective changes in their ion channel expression (Petitpré et al., 2018; Shrestha et al., 2018; Sun et al., 2018).

At many synapses such as inhibitory synapses of thalamic and cerebellar neurons, the excitatory calyx of Held synapse, and at neuromuscular junctions, the composition of VGCCs changes during development with a prominent switch from Ca_v_2.2 to Ca_v_2.1 (Siri and Uchitel, 1999; Iwasaki et al., 2000). Fast excitatory synaptic transmission in the CNS usually is accomplished by Ca_v_2.1 (Jun et al., 1999; Pagani et al., 2004; Lin et al., 2011). An increasing role for presynaptic Ca_v_2.1 in postnatal development is underlined by the fact that Ca_v_2.1^–/–^ mice die at around 3 weeks of age, when P/Q currents are essential for motoneuron function, e.g., in the respiratory system (Jun et al., 1999).

Although our data represent somatic rather than presynaptic Ca^2+^ currents, the prominent differences between N-type and P/Q-type currents of cultured α_2_δ3^+/+^ SG neurons at the two ages examined may suggest a similar developmental switch from the presynaptic Ca^2+^ channels Ca_v_2.2 at P5 to predominantly Ca_v_2.1 at P20 for the endbulb of Held synapse. If this was the case, deletion of α_2_δ3 would not only impact Ca_v_2.1-driven synaptic transmission in 3-week-old animals, but also Ca_v_2.2-driven synaptic transmission in the first postnatal week. A reduction of presynaptic N-type Ca^2+^ currents in neonatal endbulb synapses may add to the impaired morphology and diminished sizes of endbulbs observed in α_2_δ3^–/–^ mice (Pirone et al., 2014) although Ca^2+^-independent functions of α_2_δ3 in synapse formation and differentiation cannot be excluded.

Using a number of *Drosophila* mutants, Kurshan et al. (2009) showed an indispensable role of the α_2_δ3 ortholog *straightjacket* for the development of the neuromuscular junction. This novel function was observed before the Ca_v_2.1 channel ortholog *cacophony* was expressed and thus was independent of the Ca^2+^ channel function (Dickman et al., 2008; Ly et al., 2008; Kurshan et al., 2009). Notably, the α_2_δ3 ortholog *straightjacket* stabilizes the Ca_v_2.1 channel ortholog *cacophony* in *Drosophila* (Ly et al., 2008). Because α_2_δ proteins reside in the extracellular space and have protein–protein interaction domains, they may functionally interact with proteins of the extracellular matrix within the synaptic cleft or with proteins at the postsynapse. Thereby they might act as receptors for different factors involved in synaptogenesis such as thrombospondins (Resovi et al., 2014). Of note, the extracellular matrix molecules thrombospondin I and II are involved in the innervation of cochlear inner hair cells by the peripheral dendrites of SG neurons in early cochlear development (Mendus et al., 2014). While α_2_δ1 has been identified as thrombospondin receptor (Eroglu et al., 2009; Risher and Eroglu, 2012; Risher et al., 2018), interaction partners of α_2_δ3 that mediate the developmental functions of thrombospondins remain to be established.

## Ethics Statement

This study was carried out in agreement with the European Communities Council Directive (2010/63/EU) in accordance with the German law and the regional board for scientific animal experiments of the Saarland.

## Author Contributions

JE and FS conceived and designed the study. FS, VS, TE, KB, GO, and WW collected the data. FS, VS, GO, TE, KB, and JE analyzed and interpreted the data, and drafted the manuscript. JE was involved in funding acquisition and carried out the project administration.

## Conflict of Interest Statement

The authors declare that the research was conducted in the absence of any commercial or financial relationships that could be construed as a potential conflict of interest.
